# Responses of soil labile organic carbon fractions and enzyme activity in Kubuqi Desert to different vegetation restoration measures

**DOI:** 10.3389/fpls.2026.1811208

**Published:** 2026-04-17

**Authors:** Ping Zhang, Shusen Wang, Zhenqi Yang, Airong Zhou, Weifeng Wang, Yongsheng Hou, Zhihao Gao

**Affiliations:** 1 College of Desertification Control Science and Engineering, Inner Mongolia Agricultural University, Hohhot, Inner Mongolia, China; 2 State Key Laboratory of Water Engineering Ecology and Environment in Arid Area, Inner Mongolia Agricultural University, Hohhot, Inner Mongolia, China; 3 Institute of Water Resources for Pastoral Area, Ministry of Water Resources of China, Hohhot, Inner Mongolia, China; 4 Inner Mongolia Forestry Research Institute, Hohhot, Inner Mongolia, China; 5 Inner Mongolia Dalate Desert Ecosystem Observation and Research Station, Ordos, Inner Mongolia, China

**Keywords:** carbon pool management index, desertified areas, soil enzyme activity, soil labile organic carbon fractions, vegetation restoration

## Abstract

Vegetation restoration is a key strategy for reversing desertification and restoring ecosystem functions in drylands. However, its effects on soil labile organic carbon fractions, enzyme activity, and carbon pool stability in desertified areas are still largely unclear. This study focused on three typical artificial vegetation restoration measures in the Kubuqi Desert—*Caragana korshinskii* shrubs (NT), *Salix psammophila* shrubs (SL), and *Corethrodendron fruticosum* shrubs (YC)—alongside mobile sandy land. Combining field surveys and laboratory analyses, we investigated the distribution and dynamics of soil organic carbon (SOC) and its labile fractions (MBC: microbial biomass carbon, DOC: dissolved organic carbon, EOC: easily oxidizable organic carbon, LFOC: light-fraction organic carbon). We also examined the response of soil physicochemical properties and key enzyme activities, to clarify their interrelationships. Vegetation restoration measures significantly increased SOC content and storage, with NT showing the strongest effect, followed by SL and YC. Vegetation restoration measures also markedly altered the distribution of soil labile organic carbon fractions. NT significantly enhanced all measured labile carbon fractions, while YC notably increased EOC content and SL also markedly boosted LFOC. In addition, NT and SL reduced the proportion of labile carbon relative to the total SOC, indicating improved carbon pool stability. Restoration effects were pronounced in the 0–40 cm soil layer but limited at a depth of 40–60 cm. NT and SL significantly enhanced the activity of β-1, 4-glucosidase (BG), cellulobiose hydrolase (CBH), sucrase (SUC) and polyphenol oxidase (PPO), whereas YC only significantly increased SUC. Soil total nitrogen, total phosphorus, and enzyme activities were strongly correlated with labile carbon fractions and the carbon pool management index. Path analysis indicated that vegetation restoration promotes carbon transformation and sequestration by improving soil nutrient conditions and activating hydrolytic and oxidative enzyme systems. These measures drive a synergistic mechanism linking nutrients, enzymes, and carbon pools, with soil enzyme activity serving as a key indicator of carbon pool quality improvement. This study provides scientific basis for optimizing vegetation restoration strategies in desertification management and holds significant implications for assessing the soil carbon sequestration potential of ecological restoration.

## 1 Introduction

In a changing climate with more pronounced human activities, global desertification is worsening (Rivera-Marin et al., 2022). Based on the latest data from the United Nations, approximately 15.5% of the Earth’s land is degraded, representing a 4% increase compared to previous years. Desertification is expanding at an annual rate of 50, 000 to 70, 000 km², particularly in regions adjacent to deserts (Kumari et al., 2023). Desert expansion compromises the land’s biological potentia (Stiles, 1995), causing a decline in biodiversity and productivity, loss of ecological stability, and reduced carbon sequestration (Gupta, 2012). As a result, regional sustainable development is constrained, ecological security is challenged, and the living environment of hundreds of millions globally is threatened. Thus, preventing desertification and managing affected areas are strategically important for ecological security and sustainable development in arid and semi-arid regions (Chen et al., 2024). Vegetation restoration can control land degradation and is the most effective, economical, sustainable, and stable measure for mitigating desertification in arid regions (Hu et al., 2021; Li X et al., 2024; Wiesmeier et al., 2015; Le Houérou, 2000). It can reduce wind speed, block sand particles, curb desert expansion, conserve soil and water, regulate the local climate, protect water sources, and facilitate carbon sequestration, which is crucial for the sustainable development of local desert ecosystems (Jenkins and Schaap, 2018; Olschewski et al., 2018). Previous studies on vegetation restoration in desertified areas mainly focused on directly observable indicators like aboveground vegetation cover, biomass, and species diversity, and some analyzed soil physical structure and fundamental chemical properties (Han et al., 2025; Sun K. et al., 2023; Ren et al., 2022; Wu et al., 2022; Tang et al., 2018; Yang, 2022). However, the effects of such restoration on soil labile organic carbon fractions and carbon-cycle enzyme activities in these areas remain scarce.

The fundamental objective of vegetation restoration is to drive the recovery and enhancement of ecosystem functions like soil organic carbon (SOC) accumulation. SOC is a critical soil - quality indicator, and its composition and dynamics reflect the system’s carbon sequestration potential and nutrient cycling efficiency (Sun T. et al., 2023; Zhang et al., 2020). SOC is a complex of carbon fractions with different activity gradients (Lavallee et al., 2020). Variations in SOC mainly come from its readily oxidizable and rapidly cycling components (Biederbeck et al., 1994). Microbial biomass carbon (MBC), dissolved organic carbon (DOC), light-fraction organic carbon (LFOC), and easily oxidizable organic carbon (EOC) are key labile organic carbon fractions, which are biologically available carbon sources, crucial for organic carbon dynamics and nutrient cycling, and sensitive to management and environmental changes (Qu, 2022; Zhang et al., 2019). The MBC refers to the carbon present in active soil bacteria, fungi, algae, and soil microorganisms smaller than 5–105 μm³. It’s the most labile part of soil organic carbon, accounting for 1%–4% of the total, but it’s an important source and reservoir for soil nutrients, reflects soil changes, participates in soil biogeochemical transformations, and has a rapid turnover rate, playing a key role in soil fertility and plant nutrition (Moura et al., 2018; Jenkinson, 1981; Hu et al., 2012). DOC is the organic carbon that can be dissolved in water. It’s mainly composed of carbohydrates, proteins, long-chain aliphatic compounds, and macromolecular humic substances, accounting for less than 3% of soil organic carbon (Ni et al., 2003). It’s a directly available carbon source for soil microorganisms, influencing the transformation, migration, and degradation of substances in soil (Xu and Xu, 2003; Huang, 2000). Density Grading separates soil organic carbon into LFOC and heavy - fraction organic carbon. LFOC has a density below 2.0 g/cm³ (Greenland and Ford, 1964), mainly consisting of free humic acids, plant residues, and decomposition products. It’s a short-term nutrient reservoir, shows seasonal dynamics, is susceptible to environmental factors, and is affected by land use and management (Gregorich et al., 1994; Hou et al., 2011; Liu et al., 2010). It’s important for soil ecosystem structure and function and has high biological activity. EOC can be oxidized by KMnO_4_ (Blair et al., 1995). It can be rapidly oxidized and decomposed by soil microorganisms and enzymes after entering the soil with organic matter, reflecting changes in soil organic carbon (Yang et al., 2017; Chen et al., 2016). The soil EOC amount is a measure of soil fertility and SOM stability. Overall, though labile organic carbon factions make up only a small part of total SOC, they are a crucial link between vegetation inputs and the stable organic carbon pool (Cotrufo et al., 2013). Moreover, unlike total SOC with turnover times from several years to thousands of years, labile organic carbon factions have rapid turnover (usually in days, months, or years) and are extremely sensitive to management practices and environmental changes. This responsiveness, along with its close relation to microbial activity and nutrient availability, makes it a key indicator for evaluating soil functional recovery effectiveness in the later stages of vegetation restoration.

Soil enzyme activity is a key indicator of soil fertility (Wu et al., 2019; Liu et al., 2017; Fan et al., 2020; Wang et al., 2020). For example, soil β-1, 4-glucosidase (BG), Cellobiohydrolase (CBH), sucrase (SUC), polyphenol oxidase (PPO), and peroxidase (POD) are vital carbon-degrading enzymes for litter decomposition and soil organic matter mineralization (Enowashu et al., 2009; Schimel et al., 2017). BG, CBH and SUC are hydrolytic enzymes for soil carbon transformation. BG catalyzes glycosidic bond hydrolysis to produce glucose and is important for soil microorganism carbohydrate metabolism (Dick et al., 2013). SUC breaks down sucrose into glucose and fructose, and its activity reflects soil respiration and carbon conversion efficiency (Sinsabaugh et al., 2008). POD, an oxidoreductase from soil microorganisms, catalyzes oxidation and neutralizes toxic substances, playing a key role in humus formation, and its activity indicates soil respiration and microbial activity (Tian and Shi, 2014). PPO, with copper or manganese as the active center, mainly comes from soil microorganisms, plant root exudates, and residues. It catalyzes aromatic compound oxidation to form organic matter and pigments, completing the soil aromatic compound cycle (Toscano et al., 2003). Both POD and PPO are key oxidoreductases in soil carbon transformation and important ligninases (Kähkönen and Hakulinen, 2011). In summary, the levels of these soil enzyme activities not only reflect the metabolic intensity and direction of microbial communities but also exert a decisive influence on soil carbon allocation and nutrient cycling. In arid regions, where water scarcity and sparse vegetation prevail, soil microbial activity and nutrient cycling are often significantly suppressed; in this constrained environment, soil enzymes play a particularly critical regulatory role. Therefore, monitoring the activity of these soil enzymes can rapidly reveal the status of soil microorganisms and the level of nutrient cycling under arid conditions, making them indispensable indicators for assessing soil fertility and ecological restoration in arid areas.

Vegetation restoration shapes soil organic carbon and enzyme composition and function by altering vegetation inputs, microenvironments, soil physicochemical properties, and root activity (Sun et al., 2019). Previous studies across various ecosystems, including wetlands (Song et al., 2012; Shao et al., 2015), karst regions (Ha et al., 2019; Wang et al., 2023), and the Loess Plateau (Wang et al., 2020) have consistently shown that vegetation restoration increases the content of soil labile organic carbon fractions (MBC, DOC, EOC) and enhances the activity of soil enzymes (β-glucosidase, sucrase, urease, alkaline phosphatase, and amylase). Furthermore, these studies have observed significant correlations between certain soil labile organic carbon fractions and the activity of specific soil enzymes. Huang et al. (2016) also found that on gently sloping red soil peanut fields, straw-covered conservation practices increased soil DOC and MBC contents and carbon enzyme activity, and BG and cellulase activities were correlated with labile organic carbon.

In summary, characteristics of soil labile organic carbon fractions and their responses to soil enzyme activities are a hot-topic in vegetation restoration research. However, in desert vegetation restoration, systematic studies on soil labile organic carbon fraction characteristics, key soil enzyme activity dynamics, and their responses to different restoration measures are unclear. To this end, this study focused on different vegetation restoration measures (*Salix psammophila*, *Corethrodendron fruticosum*, and *Caragana korshinskii* shrubs) in the vegetation restoration area on the eastern edge of the Kubuqi Desert. By combining field surveys and laboratory analyses, it investigated the distribution and change patterns of SOC and its labile fractions (MBC, DOC, EOC, and LFOC), as well as the response patterns of soil physicochemical properties and key soil enzyme activities (BG, CBH, SUC, PPO, and POD). This study aimed to elucidate the relationships among soil physicochemical properties, enzyme activities, and labile organic carbon fractions during vegetation restoration, revealing key pathways that enhance subsurface soil function and carbon sequestration in desertified areas. We hypothesize that: (1) Compared with the control group, both SOC and labile organic carbon content will increase significantly under three vegetation restoration measures, and the effects of different measures on soil labile organic carbon will vary. (2) Vegetation restoration will increase the activity of key soil enzymes, and the effects of different measures on soil enzyme activity will vary. (3) The content of soil labile organic carbon fractions will be correlated with the activity of key soil enzymes. During vegetation restoration, changes in soil physicochemical properties drive changes in enzyme activity and the accumulation of labile organic carbon.

## 2 Materials and methods

### 2.1 Study area

**Figure 1:**
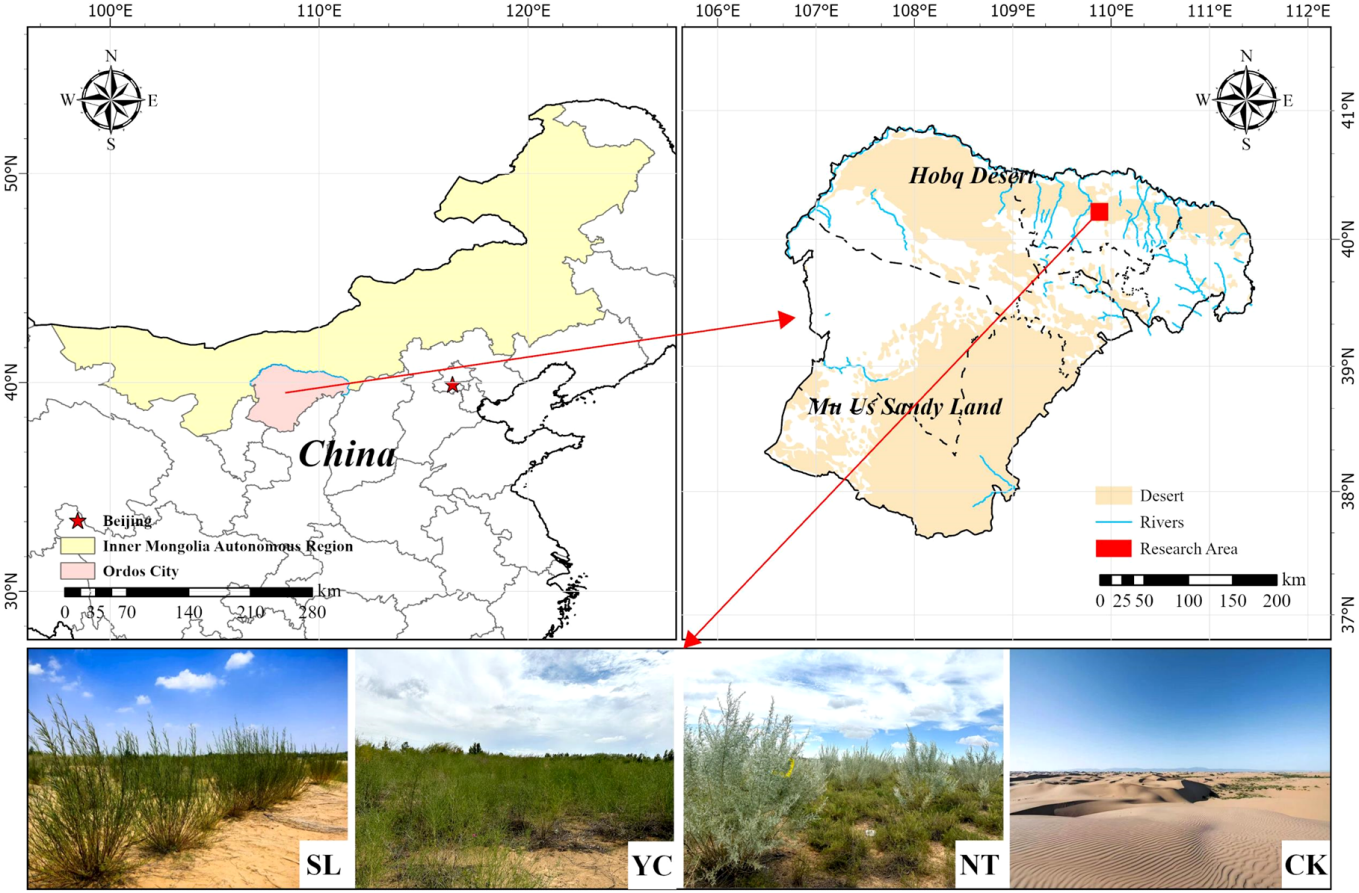
Location and basic information of the study area. SL, Salix psammophila; YC, Corethrodendron fruticosum; NT, Caragana korshinskii; CK, Mobile sandy land.

The study area is located at the eastern edge of the Kubuqi Desert, within Zhandanzhao Sumu, Ordos City, Inner Mongolia Autonomous Region, China (**Figure 1**). The climate is semi-arid temperate continental climate with cold winters, hot summers, and significant diurnal temperature variations. The average temperature in January is -17.4 °C, whereas in July, it is 33.5 °C. Annual mean temperature ranges from 6.1 to 7.1 °C, with approximately 3, 000 hours of sunshine per year and a frost-free period of 135–150 days. Annual precipitation averages 310.3 mm, concentrated from July to September. Most soils are chestnut calcareous soils and wind-blown sandy soils. Vegetation is dominated by herbaceous plants and small shrubs, including *Stipa capillata*, *Leymus chinensis*, *Agriophyllum pungens*, *Artemisia ordosica*, *Caragana korshinskii*, *Salix psammophila*, and *Corethrodendron fruticosum*.

### 2.2 Sampling plot establishment and soil sample collection

Based on preliminary surveys, three representative vegetation restoration sites, namely *Caragana korshinskii* shrubs, *Salix psammophila* shrubs, and *Corethrodendron fruticosum* shrubs, were selected within the vegetation restoration zone at the eastern edge of the Kubuqi Desert. Mobile sandy land was used as the control (**Figure 1**). All selected forest areas had been restored via planting and were located in the same climatic and soil zones, with similar elevation (1, 230–1, 260 m), slope gradient (15–20°), and aspect (90–115°E). All three forest areas had the same restoration duration of 22 years, representing the mid-to-late stages of restoration, as shown in **Table 1**.

**Table 1:** Basic information of the study area and sampling sites.

Vegetation restoration measures	Vegetation restoration tree species	Vegetation restoration methods	Restoration years	Soil types	Plant spacing	Altitude	Slope	Average plant height	AGB	Management measures
			(a)		(m)	(m)	(°)	(cm)	(kg/hm2)	
NT	*Caragana korshinskii*	Planting of seedlings	22	Aeolian sandy soil	2*10	1258	18	228.62 ± 77.11	3765.47	Enclosure management
SL	*Salix psammophila*	Planting of seedlings	22	Aeolian sandy soil	2*10	1259	16	236.52 ± 56.29	1342.38	Enclosure management
YC	*Corethrodendron fruticosum*	Aerial seeding	22	Aeolian sandy soil	–	1260	15	65.64 ± 15.57	663.40	Enclosure management
CK	Sandy land, without vegetation cover	–	–	Aeolian sandy soil	–	1230	20	–	–	–

AGB, above-ground biomass.

Soil samples were collected during the peak vegetation growth season in mid-July, 2024. Within each forest type, three standard plots measuring 20 × 20 m were randomly established, spaced more than 200 m apart. Within each plot, five sampling points were established along an “S”-shaped path. After removing surface litter, soil profiles were excavated at depths of 0–10 cm, 10–20 cm, 20–40 cm, and 40–60 cm. Samples from the same layer at each plot were thoroughly mixed, placed in a bag, sealed, and transported to the laboratory. After removing debris, the samples were homogenized and divided into two portions: one was dried naturally indoors, and the other was stored at 4 °C. The air-dried soil samples were ground and divided into two portions: one for determining SOC, EOC, and FLOC, and one for assessing the soil physicochemical properties. The refrigerated fresh soil samples were used to measure DOC, MBC, and enzyme activities.

### 2.3 Analysis

The soil pH was determined using the potentiometric method at a soil-water ratio of 1:2.5 (Sheng et al., 2018). Soil bulk density (BD) was determined using the ring knife method, and the soil water content (SWC) was measured by the oven-drying method. The soil total nitrogen (TN) content was determined using the Kjeldahl method, and the soil total phosphorus (TP) content was measured by the NaOH fusion-molybdenum antimony colorimetric method. The soil total potassium (TK) content was determined using the NaOH fusion-flame photometric method, and the soil-available phosphorus (AP) content was determined using the NaHCO_3_ extraction-molybdenum antimony spectrophotometric method. The soil-available potassium (AK) content was determined using the NH_4_OAc extraction-flame photometric method.The above methods were used to determine the soil physicochemical properties, as described by Bao (2013).

The SOC was determined using the potassium dichromate oxidation-ferrous sulfate titration method (Walkley and Black, 1934), and the DOC was measured by passing fresh soil extract through a 0.45-μm filter membrane, followed by analysis using a TOC analyzer (multi N/C3100, Analytik Jena, Germany) (Pang et al., 2019). The MBC was determined via chloroform fumigation and K_2_SO_4_ extraction (Vance et al., 1987) and the EOC via KMnO_4_ oxidation (Blair et al., 1995). LFOC was determined using the NaI density separation method. The LFOC content (mg/kg) was calculated as: LFOC content in light-fraction soil × mass fraction of light-fraction soil in total soil (Zhang et al., 2007).

Soil cellobiohydrolase (CBH) activity was determined using the p-nitrophenyl-β-D-lactoside (pNPL) colorimetric method, and soil β-1, 4-glucosidase (BG) activity was measured using the p-nitrophenyl-β-D-glucoside (pNPG) colorimetric method. Soil cellobiohydrolase (CBH) and soil β-1, 4-glucosidase (BG) activities (nmol/g/h) were defined as one enzyme activity unit (Geng et al., 2012) based on the production of 1 nmol of p-nitrophenol per gram of soil sample after incubation for 1 hour. Sucrase (SUC) activity was determined using the 3, 5-dinitrosalicylic acid colorimetric method and expressed as the amount of glucose (mg) contained in 1 g of soil over 1 day (mg/g/d) (Chen et al., 2021). Soil peroxidase (POD) activity was measured using the guaiacol colorimetric method, and polyphenol oxidase (PPO) activity was determined using the catechol colorimetric method. The activities of peroxidase (POD) and polyphenol oxidase (PPO) (mg/g/2h) were expressed as the amount (mg) of brown and red products formed in 1 g of soil after 2 hours (Ghiloufi et al., 2019).

### 2.4 Calculation of indicators

(1) The soil organic carbon storage (Tang and Li, 2013) was determined as follows:

(1) $${\text{SOC}}_{\text{storage}}=\text{SOC}\times \text{BD}\times (1-\text{θ}/100)\times \text{H}/10$$

where SOC_storage_ is the soil organic carbon storage (kg/m²); SOC is the soil organic carbon content (g/kg); BD is bulk density (g/cm³); H is soil depth (cm); θ denotes the content of gravel particles ≥ 2 mm (%), since the study area contains virtually no gravel, this value can be considered negligible.

(2) Following the methodology of Blair et al. (1995), the carbon pool management index was calculated as follows:

(2) CPMI(Carbon Pool Management Index,%)=CPI×LI×100

CPI(Carbon Pool Index)=Soil organic carbon content in the treatment group/Soil organic carbon content in the reference group

LI(Lability Index)=Lability of C in the treatment group/Lability of C in the reference group

L(Lability of Carbon)=Easyoxidated organic carbon/(Soil Organic Carbon−Easyoxidated organic carbon)

Note: The reference group soil was of moving sand soil.

### 2.5 Data analysis

Single-factor analysis of variance (ANOVA) and Duncan’s multiple range test were employed to compare significant differences among soil physicochemical properties, soil enzyme activities, soil labile organic carbon fractions, and carbon pool management indices. Differences were statistically significant at the 0.05 level (*P* < 0.05). Prior to ANOVA, normality and homogeneity of variance were assessed using the Shapiro-Wilk test and Levene’s test, respectively. All datasets met the assumptions of normality and homogeneity of variance. Using Pearson correlation analysis and redundancy analysis (RDA), we quantitatively assessed the correlations among soil physicochemical properties, enzyme activity, labile organic carbon fractions and CPMI to analyze the strength and direction of the relationships among these factors. Subsequently, to investigate the complex causal relationships among soil physicochemical properties, enzyme activity, and labile organic carbon fractions under different vegetation restoration measures, this study employed partial least squares path modeling (PLS-PM) for analysis. During model construction, vegetation restoration measures were treated as exogenous latent variables, while soil physicochemical properties (soil properties, soil nutrients), enzyme activity (soil oxidases, soil hydrolases), and labile organic carbon fractions (EOC, DOC, MBC, LFOC) were treated as mediating variables, and CPMI was treated as an endogenous latent variable. The model structure was gradually optimized, and paths with insignificant effects were removed. The PLS-PM model was plotted using the “plspm” package in R 4.5.0, and the model was comprehensively evaluated based on indicators such as the reliability and validity of the measurement model (e.g., composite reliability, AVE), the path coefficient significance (Bootstrap method), explanatory power (R²), predictive correlation (Q²), and goodness-of-fit indices (Gof) of the structural model to comprehensively evaluate the final model’s fit, ensuring its theoretical rigor and statistical validity. Statistical analyses were conducted using SPSS 26.0. Redundancy analysis was performed with the Canoco 5.0 software. Pearson correlation analysis and the figures were generated using the Origin 2021 software.

## 3 Results

### 3.1 Soil physicochemical properties under different vegetation restoration measures

**Figure 2:**
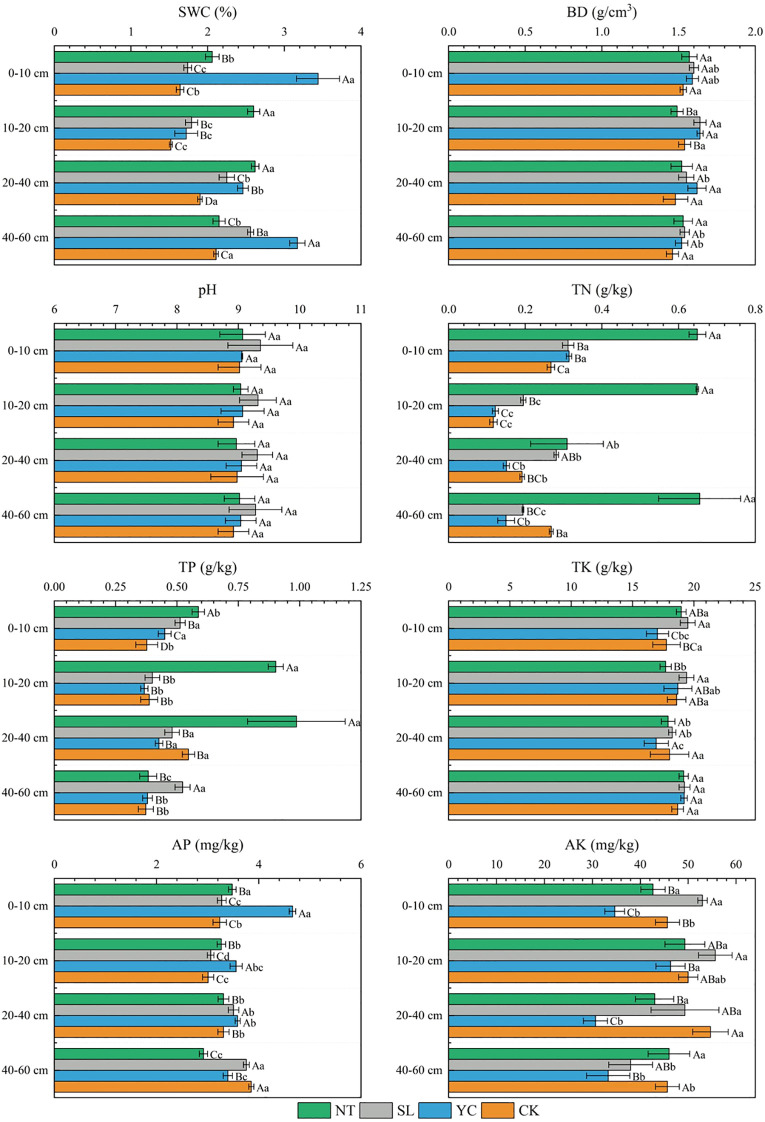
Selected soil physicochemical properties for different vegetation restoration measures. SWC, soil water content; BD, bulk density; TN, total nitrogen; TP, total phosphorus; TK, total potassium; AP, available phosphorus; AK, Available Potassium. SL, Salix psammophila; YC, Corethrodendron fruticosum; NT, Caragana korshinskii; CK, Mobile sandy land. Different capital letters indicate significant differences (*P* < 0.05) between different vegetation restoration measures in the same soil layer, and different lowercase letters indicate significant differences (*P* < 0.05) between different soil layers of the same vegetation restoration measures.

Different vegetation restoration measures and soil depths significantly influenced soil SWC, TN, TP, AP, and AK (**Figure 2**). The SWC ranged from 1.46% to 3.44%. At soil depths of 0–10- and 40–60-cm, YC exhibited the highest SWC at 3.44% and 3.17%, respectively. At depths of 10–20- and 20–40-cm, NT showed the highest SWC at 2.60% and 2.62%, respectively. Following restoration, the SWC was increased across all restoration measures compared to CK. Regarding the TN content, NT exhibited the highest values across all soil depths, ranging from 0.31 to 0.65 g/kg, significantly exceeding those of other restoration measures and CK. For the TP content, NT showed the highest levels at 0–10-cm, 10–20- cm, and 20–40-cm, ranging from 0.59 to 0.99 g/kg and significantly exceeding the levels of the other vegetation restoration measures and CK. At 40–60-cm, SL exhibited the highest TP content at 0.52 g/kg. Compared to CK, YC significantly increased the AP content in the 0–10-, 10–20-, and 20–40-cm soil layers by 1.42, 0.55, and 0.28 mg/kg, respectively. At soil depths of 0–10-cm and 10–20-cm, the AK content in the SL was highest, showing increases compared to the CK, at 53.00 mg/kg and 55.67 mg/kg, respectively. Overall, restoration significantly improved soil moisture and nutrient conditions.

### 3.2 Soil enzyme activities under different vegetation restoration measures

**Figure 3:**
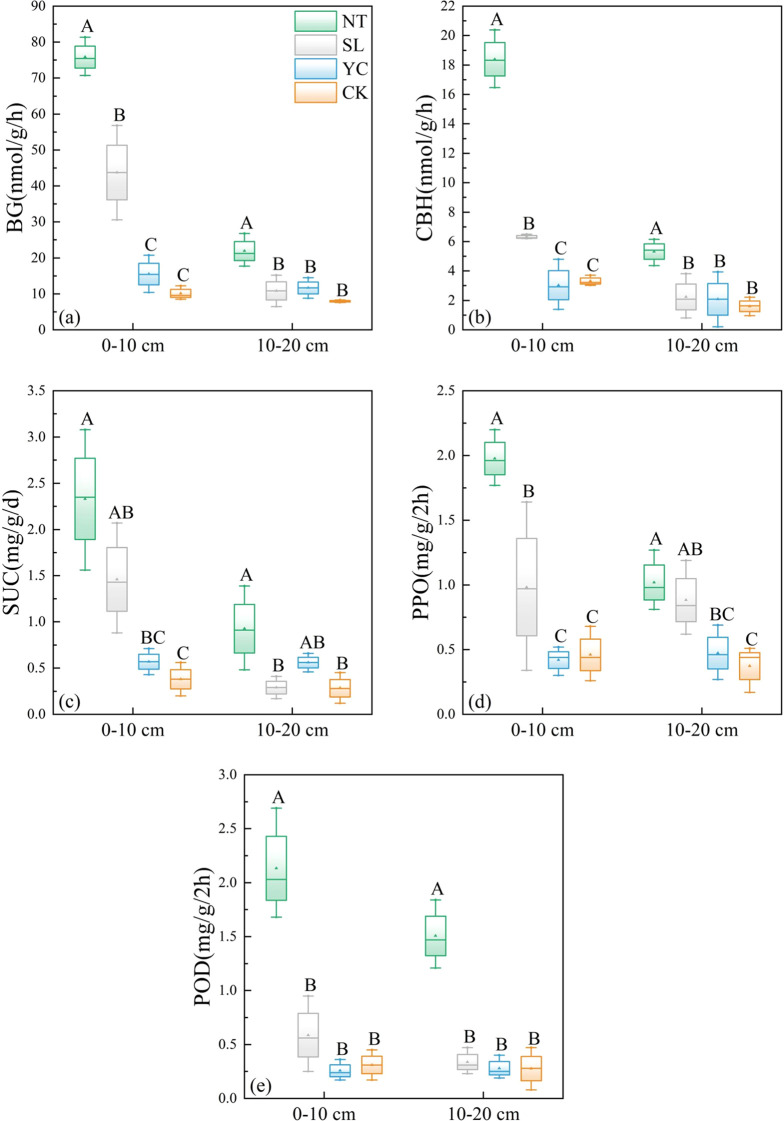
Soil enzyme activities in the different restoration measures. BG, β-1, 4-glucosidase; CBH, cellobiohydrolase; SUC, sucrase; PPO, polyphenol oxidase; POD, peroxidase; SL, Salix psammophila; YC, Corethrodendron fruticosum; NT, Caragana korshinskii; CK, Mobile sandy land. Different capital letters indicate significant differences (*P* < 0.05) among vegetation restoration measures.

The activities of all five soil enzymes, BG, CBH, SUC, PPO, and POD, differed significantly among the restoration measures. As shown in **Figure 3**, in the 0–10-cm soil layer, the activities of BG, CBH, SUC, PPO, and POD were highest in the NT, significantly higher than those in the other two vegetation restoration measures and CK, and the enhancement effect was significant compared to CK. In the 10–20-cm soil layer, the activities of the five enzymes in the NT were generally lower than those in the 0–10-cm layer, but they still maintained the relatively highest enzyme activities and showed significant differences compared to the other treatments. The BG, CBH, SUC, and PPO activities of SL were also relatively high in the 0–10-cm soil layer, second only to NT, at 43.72 nmol/g/h, 6.32 nmol/g/h, 1.46 mg/g/d, and 0.98 mg/g/2h, respectively; In the 10–20-cm soil layer, SL had a significant effect only on PPO activity, while other soil enzyme activities showed no significant difference from CK. YC had a significant effect only on SUC activity and no significant effect on other soil enzyme activities. In terms of differences across soil layers, with the exception of YC, the BG, CBH, SUC, PPO, and POD activities in the 0–10-cm soil layer of NT, SL, and CK were generally higher than those in the 10–20-cm soil layer, confirming that soil biochemical activity is primarily concentrated in the surface layer.

### 3.3 SOC content and storage under different restoration measures

**Figure 4:**
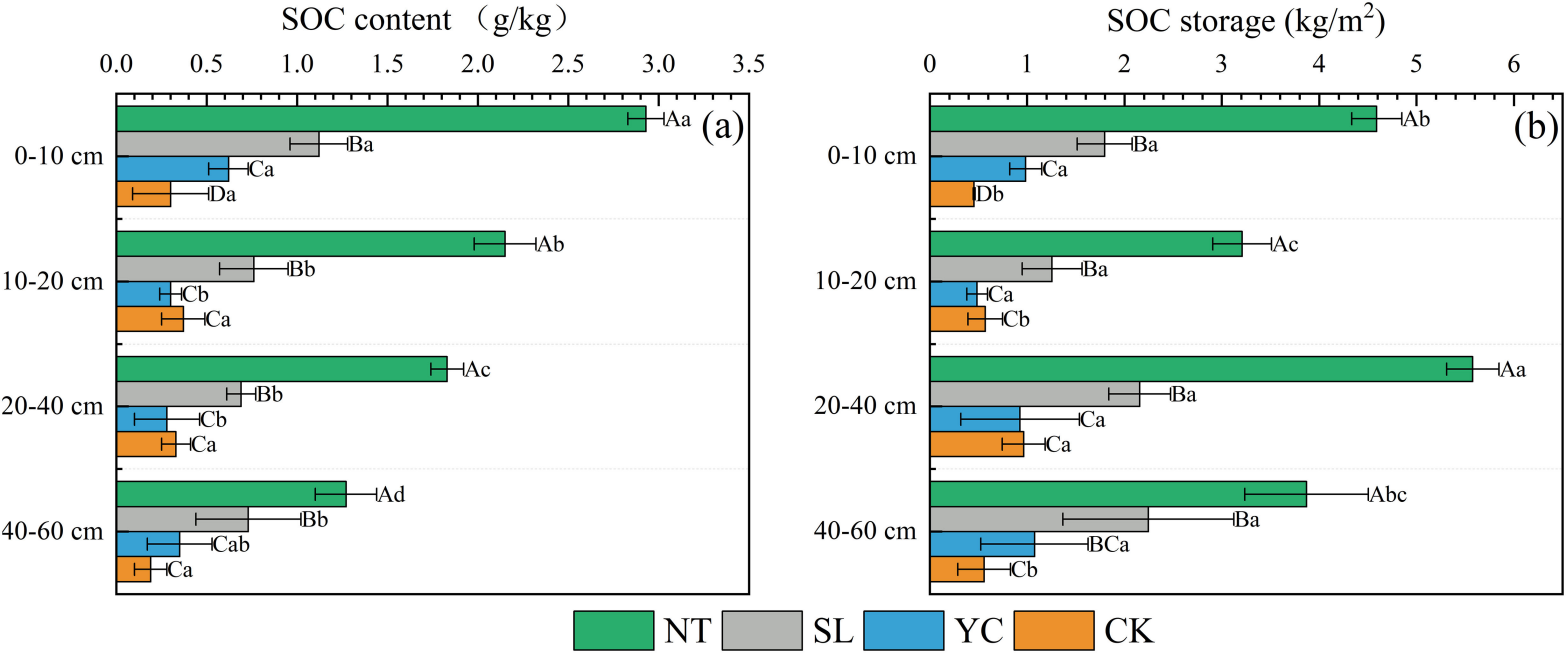
Soil organic carbon (SOC) contents **(a)** and storage **(b)** in the different restoration measures. SL, Salix psammophila; YC, Corethrodendron fruticosum; NT, Caragana korshinskii; CK, Mobile sandy land. Different capital letters indicate significant differences (*P* < 0.05) among the different restoration measures in the same soil layer. Different lowercase letters indicate significant differences (*P* < 0.05) among the different soil layers of the same restoration measures.

There were significant differences in SOC content and storage (Equation 1) between different vegetation restoration measures and CK (**Figure 4**). Among the three vegetation restoration measures, NT had the highest SOC content and storage across all soil layers, ranging from 1.27 to 2.93 g/kg and 3.21 to 5.58 kg/m², respectively, followed by SL, with SOC content and storage ranging from 0.69 to 1.12 g/kg and 1.25 to 2.24 kg/m², respectively. Both the SOC content and storage of NT and SL were significantly higher than those of YC and CK, and showed a significant increase compared to CK. YC exhibited the lowest SOC content and storage among the three vegetation restoration measures; its SOC content was significantly higher than that of CK only in the 0–10-cm soil layer, and its SOC storage was significantly higher than that of CK only in the 0–10- and 40–60-cm soil layers. In other soil layers, there were no significant differences in SOC content and storage between YC and CK. SOC content and storage in NT varied significantly across different soil layers. SOC content showed a decreasing trend with increasing soil depth, but SOC storage did not exhibit a regular pattern of change with increasing soil depth. SOC content in SL and YC was significantly higher in the 0–10-cm soil layer than in other layers, but differences among other soil layers were not significant; SOC storage in SL and YC showed no significant differences across different soil layers.

### 3.4 Soil labile organic carbon fractions and their proportions of SOC under different restoration measures

**Figure 5:**
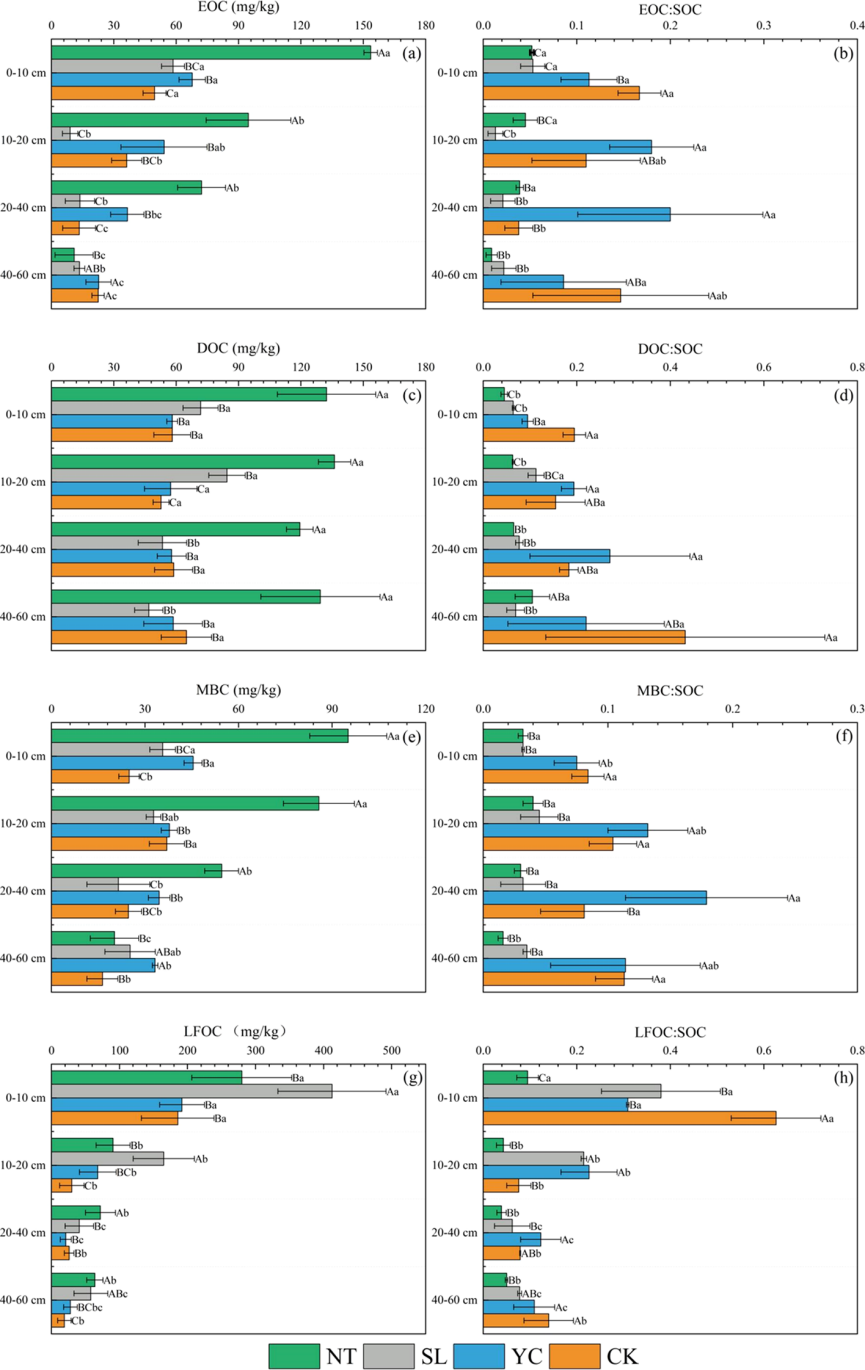
Soil labile organic carbon fraction contents **(a, c, e, g)** and their proportions **(b, d, f, h)** to total soil organic carbon in different vegetation restoration measures. SOC, soil organic carbon; MBC, microbial biomass carbon; DOC, dissolved organic carbon; EOC, easily oxidizable organic carbon; LFOC, light fraction organic carbon; SL, Salix psammophila; YC, Corethrodendron fruticosum; NT, Caragana korshinskii; CK, Mobile sandy land. Different capital letters indicate significant differences (*P* < 0.05) among the restoration measures in the same soil layer, and different lowercase letters indicate significant differences (*P* < 0.05) among the different soil layers of the same restoration measures.

The soil EOC content varied significantly among the different restoration measures and soil depths (**Figure 5a**). In the 0–10-, 10–20-, and 20–40-cm soil layers, the EOC content ranged from 72.24 to 153.38 mg/kg for NT and 36.55 to 67.71 mg/kg for YC, significantly higher than the values observed for SL and CK, with a significant increase compared to CK. Conversely, in the 40–60-cm soil layer, YC and CK exhibited higher soil EOC levels, significantly exceeding those of NT and SL. Furthermore, the EOC content in NT and YC differed significantly across the four soil layers and decreased with increasing soil depth. In contrast, in SL, most of the EOC was concentrated in the 0–10-cm layer. The EOC: SOC ratios for the three restoration measures and CK are shown in **Figure 5b**. In the 0–10- and 40–60-cm soil layers, the EOC: SOC ratios for all three restoration measures were significantly lower than those of CK. Among the three restoration measures, the EOC: SOC ratios for SL and NT were significantly lower than those for YC. Conversely, in the 10–20- and 20–40-cm soil layers, the YC treatment exhibited the highest EOC: SOC ratio, significantly exceeding those of the other two restoration treatments and CK. Except for YC, the EOC: SOC ratios for SL, NT, and CK differed significantly across the different soil layers.

As shown in **Figure 5c**, the soil DOC content under NT was highest across all soil layers, ranging from 119.49 to 136.21 mg/kg, with a marked increase when compared to that under CK. In contrast, SL only showed significantly higher values than YC and CK in the 10–20-cm soil layer. No significant differences were observed among SL, YC, and CK in the other soil layers. Furthermore, except for SL, the soil DOC content of the restoration measures and CK did not differ significantly across the different soil layers. However, there were significant differences in the DOC: SOC ratio between the different vegetation restoration measures and CK (**Figure 5d**). The CK showed the highest DOC: SOC ratio in both the 0–10- and 40–60-cm soil layers, significantly higher than the values observed for the restoration measures. However, at 10–20- and 20–40-cm, YC exhibited the highest soil DOC: SOC ratio, significantly exceeding those of CK and the other two vegetation restoration measures. The DOC: SOC ratios followed the order YC > CK > SL > NT. The soil DOC: SOC ratio for SL and NT differed significantly across the soil layers, whereas for YC and CK, there was no such difference.

There were significant differences in the MBC content among the three restoration measures and CK (**Figure 5e**). In the 0–10-cm soil layer, all three vegetation restoration measures showed significantly increased soil MBC levels when compared to those of CK, following the order NT > YC > SL. In the 10–20- and 20–40-cm soil layers, NT exhibited the highest MBC contents at 85.78 and 54.58 mg/kg, respectively. Compared with CK, NT had a significantly higher MBC content. Conversely, in the 40–60-cm layer, YC and SL showed significantly higher MBC levels compared to NT and CK. The treatment NT significantly influenced the vertical distribution of soil MBC content, which decreased with increasing soil depth. In contrast, MBC did not differ significantly across soil layers in the other two vegetation restoration measures and CK. The MBC: SOC ratio also differed significantly among restoration measures and CK (**Figure 5f**). In the 0–10-, 10–20-, and 40–60-cm soil layers, both YC and CK exhibited significantly higher MBC: SOC ratios than SL and NT. In the 20–40-cm layer, YC showed the highest MBC: SOC ratio, significantly exceeding those of the other two restoration measures and CK. The MBC: SOC ratio in YC and NT showed significant differences across soil layers, whereas no such differences were observed under SL and CK.

The LFOC levels of the different treatments and depths are shown in **Figure 5g**. In the 0–10- and 10–20-cm soil layers, SL exhibited the highest soil LFOC content at 412.38 and 165.06 mg/kg, respectively, significantly higher than those of the other two restoration measures. Compared with CK, SL had a significantly higher LFOC content. At 20–40- and 40–60-cm, NT showed the highest soil LFOC content, followed by SL, YC, and CK. Both NT and SL showed significant increases in LFOC compared to CK. Under YC, the LFOC content in the 0–10-cm layer did not differ significantly from that of CK, whereas a significant difference was observed for the other soil layers. The LFOC content in SL and YC differed significantly across the four soil layers and decreased with increasing depth. In contrast, the LFOC in NT and CK was mainly concentrated in the 0–10-cm layer. **Figure 5h** shows the LFOC: SOC ratios across the different restoration treatments and soil depths. In the 0–10-cm and 40–60-cm layers, CK showed the highest LFOC: SOC ratios (0.63 and 0.14, respectively), whereas all three restoration treatments showed lower ratios than CK, with TN showing the lowest one. However, at 10–20- and 20–40-cm, treatment YC showed the highest LFOC: SOC ratio, significantly exceeding that of the CK, whereas in NT, the value was significantly lower than that in CK. There were significant differences in the soil LFOC: SOC ratios among the three restoration treatments and the CK across the different soil layers. The LFOC: SOC ratios for SL and YC decreased with increasing soil depth. For NT and CK, the LFOC: SOC ratios were highest in the 0–10-cm layer, significantly exceeding those in the other three soil layers.

These results lead us to infer that restoration significantly increased the levels of labile organic carbon fractions in the soil. However, the effects on the ratio of labile organic carbon fractions to total organic carbon vary. For both NT and SL, the significantly decreased proportions of labile organic carbon fractions relative to total SOC indicate a compositional shift toward less labile carbon forms, suggesting a potential enhancement in carbon sink capacity. In contrast, under YC, these ratios significantly increased, enhancing the activity of SOC.

### 3.5 Carbon pool management index under different restoration measures

As shown in **Table 2**, the L values for all three restoration treatments were lower than those of CK in both the 0–10- and 40–60-cm soil layers. In CK, the L value was significantly higher than those of the three restoration measures (*P* < 0.05), following the order CK > YC > SL > NT. In the 10–20-cm soil layer, YC exhibited the highest L value at 0.22, followed by CK at 0.13. The L values for NT and SL were significantly lower than those YC and CK (*P* < 0.05). In the 20–40-cm soil layer, YC still exhibited the highest L value at 0.19, significantly higher than those of the other two restoration treatments and CK (*P* < 0.05). No significant differences were observed between the other two vegetation restoration measures and CK. Overall, both NT and SL reduced the lability of soil carbon.

CPMI (Equation 2) is a comprehensive indicator for quantitatively evaluating the impact of management practices on the quality of soil organic carbon pools. Its core principle lies in assessing the effectiveness of management practices by comparing the organic carbon status of managed soils with that of reference soils, with the reference soil’s CPMI value defined as 100%. In the 0–10-, 10–20-, and 20–40-cm soil layers, NT exhibited the highest CPMI values of 274.44%, 263.62%, and 687.79%, respectively. The CPMI values followed the order NT > YC > SL, with all three measures exceeding 100%. In the 40–60-cm soil layer, YC exhibited the highest CPMI at 95.24%, followed by SL at 54.04%, whereas NT had the lowest value at 41.13%. The CPMI values for all three vegetation restoration measures were below 100%.

**Table 2:** Soil carbon pool management index (CPMI) values and related indices in different restoration measures.

Indices	Soil depth	NT	SL	YC	CK
	(cm)				
L	0–10	0.06 ± 0.00Ca	0.06 ± 0.01Ca	0.13 ± 0.04Bab	0.20 ± 0.03Aa
	10–20	0.05 ± 0.01BCa	0.01 ± 0.01Cb	0.22 ± 0.07Aa	0.13 ± 0.08Bab
	20–40	0.04 ± 0.01Ba	0.02 ± 0.01Bb	0.19 ± 0.10Aab	0.04 ± 0.02Bb
	40–60	0.01 ± 0.01Bb	0.02 ± 0.01Bb	0.08 ± 0.04ABb	0.18 ± 0.14Aa
LI	0–10	0.28 ± 0.04Bb	0.29 ± 0.12Bb	0.63 ± 0.08Aa	
	10–20	0.52 ± 0.42ABb	0.10 ± 0.01Bc	2.40 ± 1.66Aa	
	20–40	1.13 ± 0.41Aa	0.50 ± 0.13Aa	6.38 ± 5.75Aa	
	40–60	0.07 ± 0.02Bb	0.14 ± 0.02Bbc	0.52 ± 0.12Aa	
CPI	0–10	9.86 ± 0.34Aa	3.78 ± 0.64Ba	2.08 ± 0.31Ca	
	10-20	6.35 ± 2.61Aa	2.09 ± 0.17Bb	0.83 ± 0.19Bb	
	20–40	5.81 ± 1.25Aa	2.26 ± 0.86Bb	0.81 ± 0.35Bb	
	40–60	7.56 ± 2.98Aa	3.96 ± 0.42Ba	1.84 ± 0.09Ba	
CPMI	0–10	274.44 ± 32.45Ab	105.92 ± 26.20Ba	128.30 ± 1.87Bb	
	10–20	263.62 ± 126.14Ab	101.69 ± 3.76Ba	183.24 ± 116.49ABab	
	20–40	687.79 ± 396.05Aa	105.66 ± 14.74Ba	386.99 ± 194.59ABa	
	40–60	41.13 ± 32.41Bb	54.04 ± 14.87ABb	95.24 ± 19.25Ab	

L, Lability of carbon; LI, Lability index; CPI, Carbon pool index; CPMI, Carbon pool management index; SL, Salix psammophila; YC, Corethrodendron fruticosum; NT, Caragana korshinskii; CK, Mobile sandy land. Different capital letters indicate significant differences (*P* < 0.05) among different restoration measures in the same soil layer; different lowercase letters indicate significant differences (*P* < 0.05) among different soil layers of the same restoration measures.

### 3.6 Relationship between soil labile organic carbon fractions, physicochemical properties, and enzyme activity

**Figure 6:**
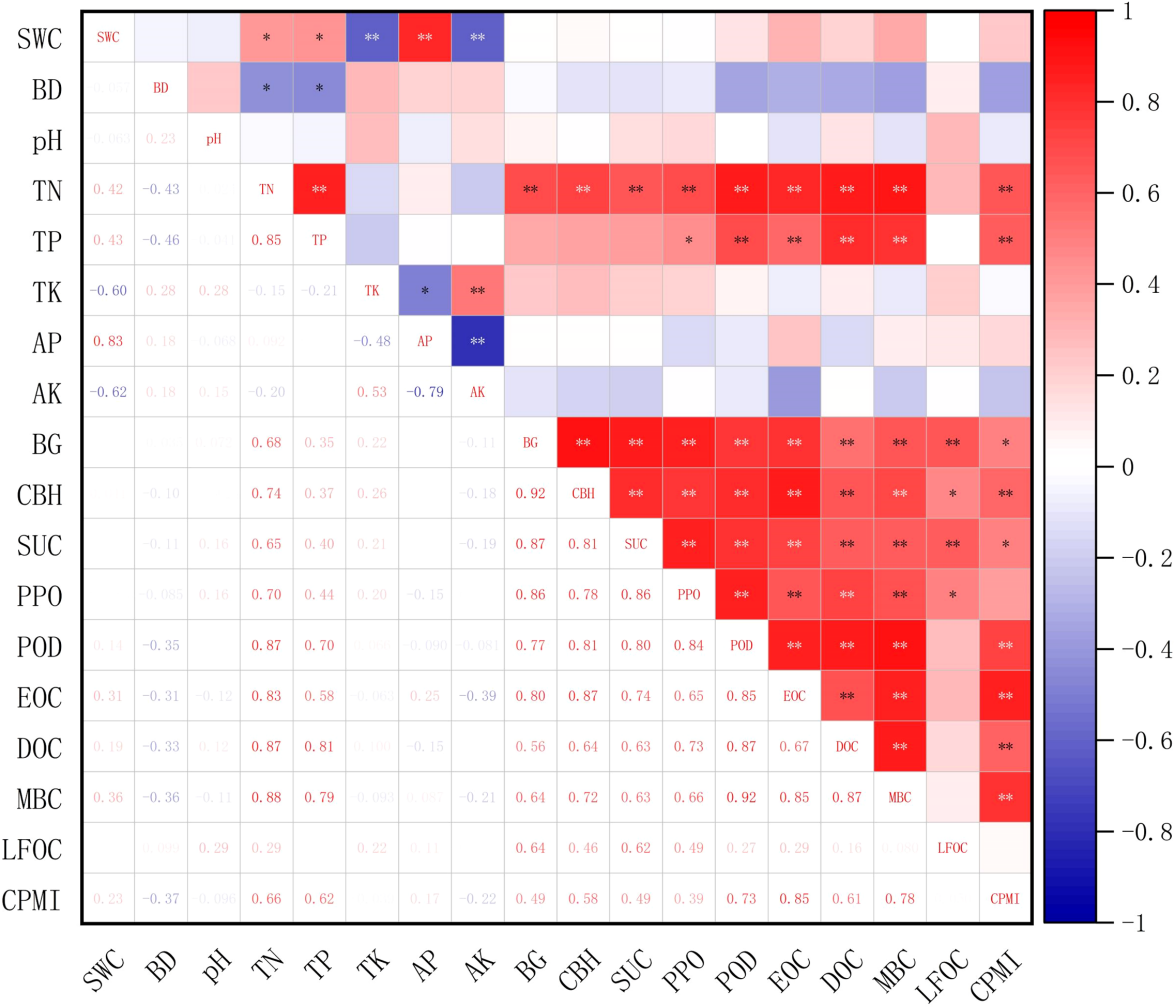
Correlations between soil physicochemical properties with soil enzyme activities and soil labile organic carbon fraction contents. SWC, soil water content; BD, bulk density; TN, total nitrogen; TP, total phosphorus; TK, total potassium; AP, available phosphorus; AK, Available Potassium; BG, β-1, 4-glucosidase; CBH, cellobiohydrolase; SUC, sucrase; PPO, polyphenol oxidase; POD, peroxidase; MBC, microbial biomass carbon; DOC, dissolved organic carbon; EOC, easily oxidizable organic carbon; LFOC, light-fraction organic carbon; CPMI, carbon pool management index. *, ** represent significant (*P* < 0.05) and highly significant (*P* < 0.01) correlations, respectively.

As shown in **Figure 6**, EOC, DOC, and MBC were highly significantly correlated (*P* < 0.01) with TN, TP, BG, CBH, SUC, PPO, and POD. Further, LFOC showed highly significant positive correlations with BG and SUC (*P* < 0.01) and significant positive correlations with CBH and PPO (*P* < 0.05). The CPMI was highly significantly correlated with TN, TP, CBH, and POD (*P* < 0.01) and significantly positively correlated with BG and SUC (*P* < 0.05). Additionally, TN was highly significantly positively correlated with BG, CBH, SUC, PPO, and POD (*P* < 0.01), and TP was highly significantly correlated with POD (*P* < 0.01) and significantly positively correlated with PPO (*P* < 0.05).

**Figure 7:**
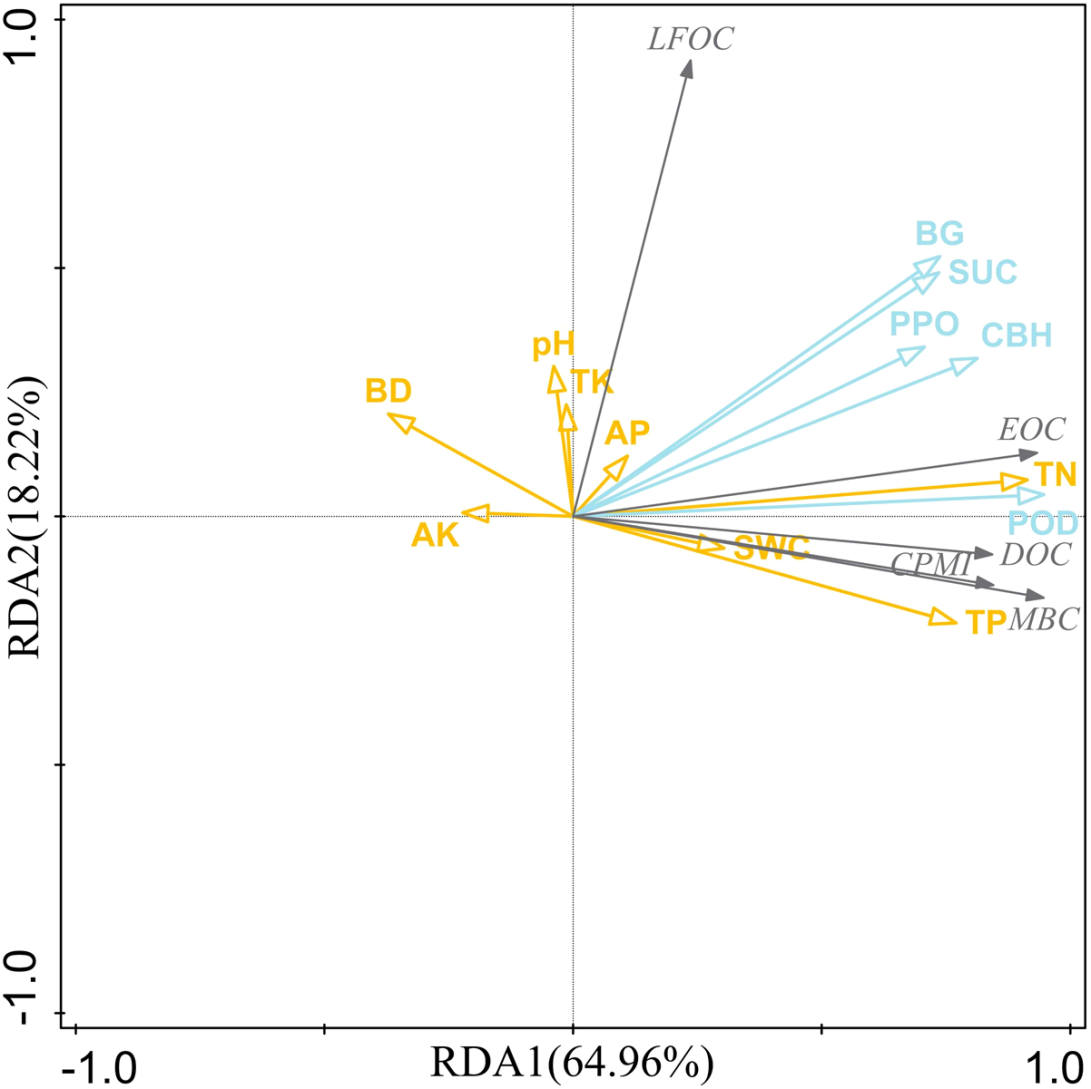
Results of the redundancy analysis of soil labile organic carbon fraction with soil physicochemical properties and soil enzyme activities. SWC, soil water content; BD, bulk density; TN, total nitrogen; TP, total phosphorus; TK, total potassium; AP, available phosphorus; AK, Available Potassium; BG, β-1, 4-glucosidase; CBH, cellobiohydrolase; SUC, sucrase; PPO, polyphenol oxidase; POD, peroxidase; MBC, microbial biomass carbon; DOC, dissolved organic carbon; EOC, easily oxidizable organic carbon; LFOC, light-fraction organic carbon; CPMI, carbon pool management index. The gray line indicates the response variable, and the blue and yellow line indicates the explanatory variable.

The RDA results in **Figure 7** indicate that the soil physicochemical properties and enzyme activities explained 83.18% of the total variance in labile organic carbon fractions and CPMI. Soil physicochemical properties and enzyme activities contributed 93.20% to the variance in soil labile organic carbon fractions and CPMI (**Table 3**). Among these, POD, BG, and PPO were the main influencing factors, contributing 62.80%, 12.40%, and 7.60% to the variance in soil labile organic carbon fractions and CPMI, respectively.

**Table 3:** Interpretation rates of the single environmental factors in redundancy analysis.

Soil environment factor	Explains (%)	Contribution(%)	*P*-value
POD	58.5	62.8	0.002
BG	11.5	12.4	0.002
PPO	7.1	7.6	0.004
CBH	3.4	3.7	0.008
SWC	1.8	1.9	0.048
AK	2.5	2.7	0.066
TN	2.3	2.4	0.092
SUC	2	2.1	0.13
TP	1.8	1.9	0.118
BD	0.9	0.9	0.26
AP	0.9	1	0.298
pH	0.3	0.3	0.706
TK	0.2	0.2	0.858

SWC, soil water content; BD, bulk density; TN, total nitrogen; TP, total phosphorus; TK, total potassium; AP, available phosphorus; AK, Available Potassium; BG, β-1, 4-glucosidase; CBH, cellobiohydrolase; SUC, sucrase; PPO, polyphenol oxidase; POD, peroxidase.

**Figure 8:**
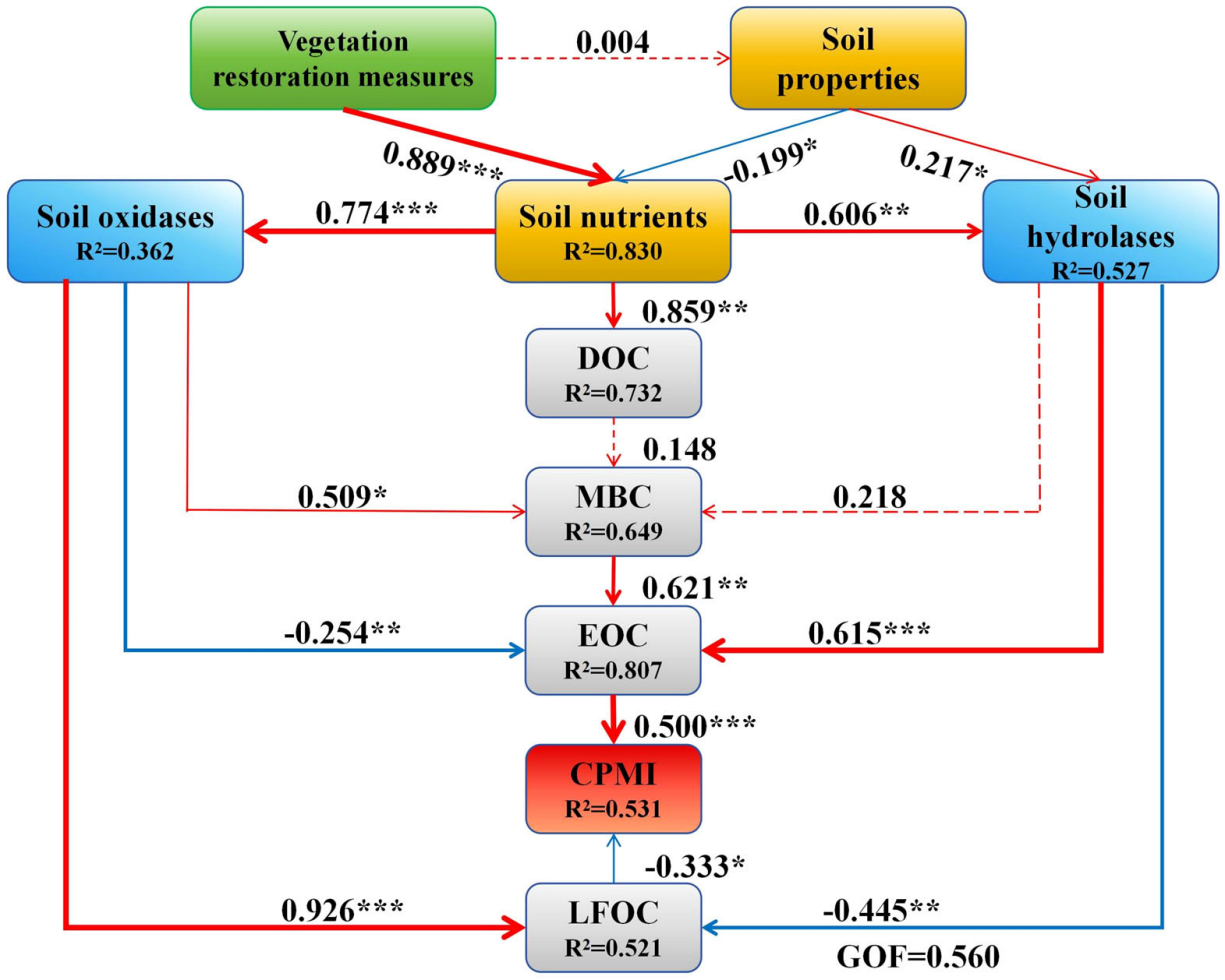
Results of the partial least squares path model (PLS-PM). Red and blue arrows indicate positive and negative effects (*P* < 0.05), respectively. The dotted line arrow indicates that the effect is not significant. The numbers on the arrows represent standardized path coefficients. The width of the arrows is proportional to the strength of the path coefficients. Vegetation types: *Salix psammophila* (SL), *Corethrodendron fruticosum* (YC), *Caragana korshinskii* (NT). GOF: Goodness of fit. *P < 0.05, **P < 0.01, ***P < 0.001.

We employed PLS-PM to determine the direct and indirect relationships between soil labile organic carbon fractions and CPMI with soil physicochemical properties and enzyme activities, explaining 73.20%, 64.90%, 80.70%, 52.10%, and 53.10% of the variance, respectively (**Figure 8**). Restoration measures had a significant positive effect on soil nutrients (*P* < 0.001). Soil nutrients directly influenced DOC positively (*P* < 0.01) and indirectly affected MBC, EOC, LFOC, and CPMI through direct positive effects on soil oxidase and soil hydrolase. Among these, soil oxidase significantly positively affected MBC and LFOC while significantly negatively affecting EOC (*P* < 0.01) and indirectly influencing CPMI. Soil hydrolytic enzymes significantly positively affected EOC (*P* < 0.001) and significantly negatively affected LFOC (*P* < 0.01), indirectly influencing CPMI. The EOC positively influenced CPMI (*P* < 0.001), whereas LFOC negatively affected CPMI (*P* < 0.05). Among all factors evaluated in this study, soil oxidases (PPO and POD) and hydrolytic enzymes (BG, CBH, and SUC) had the most significant effects on soil active organic carbon fractions and CPMI, followed by soil nutrients (e.g., TN, TP).

## 4 Discussion

### 4.1 Effects of vegetation restoration on SOC and labile fractions

The SOC content is governed by a dynamic balance between carbon inputs from plant residues, root exudates, and root biomass, and carbon losses through soil respiration and erosion-driven transport. Its accumulation is influenced by a combination of factors including soil development, vegetation type, and human disturbance (Laganière et al., 2010). In this study, following vegetation restoration, SOC content and storage increased across all restoration measures compared to the control. Specifically, NT showed average increases of 597.71% and 612.03% in soil organic carbon content and reserves, respectively; SL showed average increases of 194.41% and 210.48%, respectively; YC recorded average increases of 40.45% and 47.72%, respectively. Previous studies have also demonstrated that vegetation restoration significantly increases organic carbon content (Yang et al., 2016), with reforestation leading to increases of 35–100% in organic carbon content (Johnson, 1992). The reasons for the significant differences in SOC content and storage between vegetation restoration measures and the control are as follows: first, vegetation restoration can enrich surface flora and fauna communities, increase inputs of soil organic matter (such as litter and animal residues), and promote the accumulation of soil organic carbon (Huang et al., 2012). Vegetation restoration improves litter, root system coefficients, root exudates, microbial activity, and soil structure, accelerating the accumulation of soil organic carbon. Second, after vegetation restoration in sandy areas, ground cover increases. Shrub canopies reduce near-surface wind speeds, slow runoff velocity and sediment load, significantly reducing soil organic carbon loss from wind and water erosion, thereby further enhancing soil organic carbon accumulation. Additionally, the study also found significant differences in SOC content and storage among different vegetation restoration measures, with the following ranking: NT > SL > YC. This is because SOC is influenced by vegetation return and decomposition rates. High vegetation return and rapid decomposition rates lead to rapid SOC accumulation. As a deep-rooted leguminous shrub, *Caragana korshinskii* enhance soil nitrogen through their nitrogen-fixing capacity, which directly promotes the accumulation, growth, and reproduction of soil microorganisms. This indirectly facilitates the sequestration and storage of SOC (Imbufe et al., 2005). Second, the extensive woody root biomass of *Caragana korshinskii* can deliver substantial organic carbon to deeper soil layers. Its litter possesses high lignin content and decomposes relatively quickly, facilitating soil organic carbon accumulation. Although *Salix psammophila* is a non-leguminous shrub, its rapid growth and large biomass enable significant carbon input through abundant litter and root exudates. However, *Salix psammophila* litter typically decomposes more slowly than *Caragana korshinskii* litter, potentially contributing less to soil organic carbon replenishment (Guo et al., 2014). Although *Corethrodendron fruticosum*, as a leguminous subshrub or small subshrub, possesses nitrogen-fixing capabilities, its aboveground and root biomass are typically lower than those of the former two, resulting in relatively low initial carbon input. This is the fundamental reason for its weaker capacity to accumulate soil organic carbon.

Soil labile organic carbon can regulate the availability of organic matter and nutrients in soil and is highly sensitive to environmental changes, making it suitable for evaluating early changes in soil quality (Woldeselassie et al., 2012; Hu and Lan, 2020; Wang et al., 2020). In the present study, restoration significantly altered the levels of DOC, MBC, EOC, and LFOC in the soil, and the different restoration measures differed in their impacts on labile organic carbon fractions. NT had the most pronounced positive effects on EOC, DOC, and MBC, whereas SL had the greatest positive effect on the LFOC content. In contrast, YC showed lower values of DOC, MBC, and LFOC. This can be explained as follows: differences in vegetation types and habitat conditions influence the responses of soil labile organic carbon fractions to restoration measures. On the one hand, *Caragana korshinskii* forms a symbiotic nitrogen-fixing relationship with rhizobia. This process consumes a significant amount of photosynthetic products, prompting the root system to secrete more labile carbon sources such as organic acids and sugars, which directly increases the levels of DOC and EOC in the soil. Furthermore, the abundant rhizosphere exudates and easily degradable litter provide high-quality energy sources for microorganisms, significantly stimulating their growth and reproduction; consequently, the MBC content also increases substantially. Compared to *Caragana korshinskii*, *alix psammophila* has a higher cellulose content and a relatively moderate degree of lignification, which facilitates gradual breakdown into light fraction matter under microbial action rather than rapid mineralization or conversion into compact humus. Consequently, *alix psammophila* has a higher LFOC content. In contrast, the limited biomass input from *Corethrodendron fruticosum* results in weaker stimulation of microbial community size and activity, leading to a relatively slower turnover rate of labile organic carbon. On the other hand, the DOC mainly originates from root exudates and plant litter leachates, making it susceptible to soil leaching (Pang et al., 2019). In this study, SL and YC lacked abundant surface litter and well-developed root systems. Furthermore, sandy soils, lacking physical protection for input organic carbon (Richter et al., 1999), tend to leach DOC from the soil. However, organic matter input into the NT topsoil layer could counteract this leaching effect.

The levels of organic carbon and some of its labile fractions generally decreased with increasing soil depth, consistent with previous findings (Hu and Lan, 2020; Wang et al., 2020, 2023). This can be explained by the higher humus content in topsoil compared to subsoil (Wang et al., 2014). Organic matter input promotes the growth and reproduction of soil microorganisms, thereby facilitating the accumulation of SOC and its labile fractions (Schlesinger, 1990; Shi et al., 2023). The topsoil is also the main distribution zone for plant roots. With increasing soil depth, both organic matter input and belowground biomass decrease significantly, leading to reduced levels of soil organic carbon and its labile fractions (Barreto et al., 2011; Fontaine et al., 2007).

### 4.2 Effects of vegetation restoration on soil carbon pool management index

Carbon lability is an indicator of the ratio between the instability and stability of SOC (Li Y et al., 2024, 2025). In this study, the L values for the three restoration measures ranged from 0.01 to 0.22, significantly lower than those reported by Pu et al. (2017) for three restoration models in the desertified grasslands of Ruoergai (0.35–2.05) and by Li et al. (2012) for the topsoil (0–20 cm) of wasteland and pine forest under different land-use practices (0.44 and 0.45, respectively). Furthermore, both NT and SL exhibited lower L values than CK across all soil layers, reducing the activity of the SOC pool and enhancing its stability, which is of importance in desert regions. These differences can be attributed to the combined effects of the study area’s unique environmental context, vegetation types, and soil properties. First, compared to the mobile sandy land with no stable carbon inputs, the restored vegetation in *Caragana korshinskii* and *Salix psammophila* shrubs typically produces litter (such as dead branches and roots) with higher carbon-to-nitrogen ratios (C/N) and lignin content. These organic materials have complex molecular structures that resist rapid microbial decomposition, qualifying them as “inert” or “stable” inputs. Consequently, whilst they increase the total SOC stocks, the added carbon mainly occurs in slow-turnover forms, lowering the average activity of the entire soil carbon pool. Second, as an ecologically fragile region, the Kubuqi Desert features inherently poor soils with scarce moisture. Both *Caragana korshinskii* and *Salix psammophila* are deep-rooted, drought-tolerant shrubs whose litter decomposes slowly and releases carbon at low rates. This results in prolonged SOC turnover cycles and a small proportion of labile fractions, leading to low carbon pool lability values. Overall, vegetation restoration in desert regions facilitates the transformation of carbon in more stable forms, thereby enhancing carbon pool stability.

CMPI is an indicator characterizing the relationship between the total SOC pool and its active components, reflecting the potential for SOC sequestration and mineralization in soil (Yang et al., 2018). Following restoration, CPMI values exceeding 100% indicate an increased SOC sequestration capacity and a favorable soil development (Benbi et al., 2015; Blair et al., 1995). In this study, the CPMI values varied across soil layers under different vegetation restoration measures. For the 0–40-cm soil layer, the CPMI exceeded 100% under all restoration treatments, indicating that compared to the control, each restoration measure improved soil quality, albeit to varying degrees. This finding aligns with previous research (Xiao et al., 2025; Qiu et al., 2011; Gong et al., 2008). Concurrently, due to variations in biomass, root distribution, and litterfall quantity among the different restoration measures, their capacity to enhance soil quality followed the order NT > SL > YC. However, for all vegetation restoration measures, the CPMI remained below 100% at 40–60-cm. This indicates that ecological restoration in sandy areas can significantly enhance the SOC sequestration capacity of surface and shallow soils but has little effect on deep soils, most likely because plants contribute to the soil carbon pool mainly through litter return and root activity.

Litter decomposition, fine root turnover, and root exudates are concentrated in the surface and shallow soil layers (typically 0–40-cm), which represent the most biologically active zone with the highest rate of organic matter transformation. Therefore, following restoration, plants significantly increase the input and sequestration of active organic carbon in this layer through their extensive root networks and vigorous physiological activity, thereby substantially elevating the CMPI. However, in the deeper soil layer (40–60-cm), plant roots are sparse, limiting organic carbon input. This layer’s carbon pool primarily relies on the slow and inefficient migration of organic matter from the upper layers via leaching. Additionally, the loose structure and large pores of sandy soils are unfavorable for the adsorption and physical protection of DOC, making it difficult for any restoration measures to significantly impact deep soil carbon pools. Consequently, the ameliorative effect of restoration on soil carbon pools in sandy areas exhibits pronounced surface-layer dominance.

### 4.3 Effects of vegetation restoration on soil enzyme activity

Soil enzyme activity refers to the capacity to catalyze biochemical reactions in soil and is an indicator of the activity of soil microbial populations and, consequently, soil quality and health (Liu et al., 2017; Wu et al., 2019). It is sensitive to changes in land cover, which can cause significant alterations in the labile organic carbon fractions of soil, ultimately leading to changes in the soil carbon pool (Burns et al., 2013). Soil enzymes such as BG, CBH, SUC, PPO, and POD are closely associated with soil organic carbon accumulation and mineralization (Schimel et al., 2017). They regulate the biochemical processes governing the formation and decomposition of soil labile organic carbon fractions and serve as important indicators of soil fertility (Fan et al., 2020; Wang et al., 2020). In our study, both NT and SL increased the activity of the five soil enzymes to varying degrees, but the difference between YC and CK was not significant. The effects of different restoration measures on soil enzyme activity were not entirely consistent. As demonstrated, following restoration in sandy areas, the BG, CBH, SUC, PPO, and POD activities can be significantly enhanced. Under different vegetation restoration measures, the soil enzyme activities vary due to differences in vegetation type, litter, root exudates, and soil microbial abundances (Liu et al., 2016). Additionally, in this study, the activities of most soil enzymes in NT, SL, and CK were higher in the 0–10 cm soil layer than in the 10–20 cm layer. Similar results have been observed elsewhere (Shen et al., 2024; Wang et al., 2020; Chen et al., 2016). This can be explained by the fact that the topsoil harbors a greater concentration of enzyme-releasing organisms, along with abundant nutrient sources and favorable temperature, water, and aeration conditions (Liu et al., 2019; Stone et al., 2014; Chen et al., 2019). With increasing soil depth, the organic matter content sharply declines, limiting microbial growth, and, consequently, reducing soil enzyme activity (Xu et al., 2020; Eilers et al., 2012).

In desert soils, microorganisms allocate significant resources to osmotic stress tolerance, limiting enzyme production. Vegetation restoration alleviates this stress by improving soil moisture and organic matter inputs, enabling microbes to reallocate resources toward enzyme synthesis. The concurrent increases in both labile carbon substrates and the enzymes that process them suggest a positive feedback loop consistent with functional recovery, rather than merely a passive response to higher substrate availability. Differential responses among restoration measures may reflect shifts in microbial resource allocation strategies: under NT, microbes likely adopt a “resource acquisition” strategy investing in hydrolytic enzymes, while under SL, a “nutrient mining” strategy favors oxidative enzymes to access nutrients in recalcitrant litter. These findings suggest that restoration not only increases enzyme activities but also shifts microbial adaptive strategies, contributing to soil functional recovery.

### 4.4 Key factors affecting soil labile organic carbon fractions during vegetation restoration

The soil labile organic carbon fractions (EOC, DOC, MBC, LFOC) and CPMI were significantly positively correlated with soil nutrients (e.g., TN, TP) and carbon cycle-related enzyme activities (BG, CBH, SUC, PPO, POD) (*P* < 0.01), indicating that soil nutrient accumulation and enhanced soil enzyme activity jointly promote the formation and transformation of labile organic carbon. Redundancy analysis (RDA) further validated that soil physicochemical properties and enzyme activities collectively explained 83.18% of the variation in labile organic carbon fractions and CPMI, with POD, BG, and PPO being the dominant factors that contributed 62.8%, 12.4%, and 7.6%, respectively. This indicates that in arid sandy regions, oxidase and hydrolase synergistically regulate organic carbon transformation efficiency, making them core factors that improve carbon pool quality.Mechanism analysis based on PLS-PM indicates that restoration measures indirectly influence labile organic carbon fractions and CPMI by directly regulating soil nutrients (path coefficient: 0.889, *P* < 0.001). Further, soil nutrients exert a direct positive effect on DOC (*P* < 0.01) and indirectly regulate the accumulation of MBC, EOC, and LFOC by stimulating the activity of hydrolytic enzymes (BG, CBH, SUC) and oxidizing enzymes (PPO, POD). Among these, hydrolytic enzymes primarily promote EOC formation (path coefficient: 0.615, *P* < 0.001), whereas oxidizing enzymes have a significant positive effect on MBC and LFOC (*P* < 0.01) but a negative effect on EOC (path coefficient: -0.254, *P* < 0.01). These findings suggest that in sandy soils, the accumulation of readily decomposable carbon fractions (e.g., EOC) relies more on the rapid mineralization of sugars driven by hydrolytic enzymes, whereas oxidases may promote the formation of stable carbon pools by catalyzing the polymerization of aromatic compounds. The opposite contributions of EOC and LFOC to the carbon pool management index (CPMI), positive for EOC and negative for LFOC, further indicate that the chemical properties and turnover rates of labile carbon fractions govern the direction of CPMI responses.

The impact intensity of different restoration measures is closely related to litter mass, root distribution, and microbial communities. *Caragana korshinskii* and *Salix psammophila*, due to their higher biomass input and well-developed root systems, significantly increased the levels of soil TN, TP, and enzyme activity, thereby promoting the accumulation of labile organic carbon and enhancing the CPMI. In contrast, *Corethrodendron fruticosum* showed less pronounced effects on soil nutrients and enzyme activity due to its slower litter decomposition rate and limited root input, resulting in a lower carbon pool activation. This finding aligns with observations reported by Wang et al. (2023) and Shen et al. (2024) for karst ecosystems, indicating that vegetation types drive carbon pool differentiation by regulating the “nutrient-enzyme-carbon” synergistic pathway.

From the above analysis, we can see that soil microorganisms (represented by MBC) act as a bridge in the relationship between soil labile organic carbon fractions and soil enzyme activity. DOC is the most readily available energy source for microorganisms; its accumulation provides an abundant substrate for microorganisms, leading to an increase in the MBC pool. An active microbial community synthesizes and secretes more enzymes (including BG, SUC, and CBH) to meet its metabolic demands. The high activity of hydrolytic enzymes such as BG, SUC, and CBH further accelerates organic matter decomposition, generating more labile organic carbon fractions (e.g., EOC). The involvement of oxidases (PPO, POD) suggests that, under conditions of abundant labile carbon, the system may simultaneously initiate decomposition strategies targeting refractory carbon pools to maintain long-term carbon and energy supply. PPO and POD are primarily involved in the oxidation of complex, refractory organic matter such as lignin. When labile carbon sources (e.g., DOC, EOC) are abundant, microbial communities may allocate more resources to synthesizing oxidases such as PPO and POD. During the mid-stage of vegetation restoration, with the influx of large amounts of litter, the increased activity of oxidases may primarily serve to process the continuously accumulating lignin. The direct products of this process (partially degraded, not yet fully humified organic fragments) constitute the main components of LFOC, thereby leading to an increase in LFOC content.

In summary, vegetation restoration measures has an impact on CPMI by altering soil nutrient conditions, activating hydrolytic and oxidative enzyme systems, and, consequently, regulating the dynamic equilibrium of labile organic carbon fractions. As a key biological indicator linking vegetation inputs to carbon pool stability, soil enzyme activity warrants significant attention in future assessments of the efficiency of ecological restoration efforts in arid regions.

Limitations of this study: (1) This study is primarily based on samples collected at a single point in time and thus fails to reveal the dynamic patterns of soil labile organic carbon fractions and enzyme activity as they change over the years of restoration. The dominant factors influencing carbon pool accumulation rates and enzyme activity may vary across different stages of restoration; the lack of time-series data limits our understanding of the long-term trends and mechanisms underlying the restoration process. (2) Soil biochemical processes are significantly influenced by water and thermal conditions. Since this study did not collect samples across different seasons, it was unable to assess the effects of seasonal factors such as temperature and humidity on soil labile organic carbon fractions and soil enzyme activity. This may obscure the true strength of the relationship between enzyme activity and carbon fractions or overlook their driving role during critical periods of nutrient cycling. (3) While this study systematically measured soil physicochemical properties and enzyme activity, it did not analyze the soil microbial community (e.g., the composition, diversity, and functional genes of bacteria and fungi). Microorganisms are the primary agents of soil organic carbon transformation and enzyme secretion; the lack of microbial analysis makes it difficult for this study to elucidate the underlying biological mechanisms by which different vegetation types influence soil carbon processes.

## 5 Conclusion

Vegetation restoration measures can significantly alter the distribution and accumulation of SOC pools and the labile organic carbon fractions in desertified areas, with the effects varying by vegetation type. All vegetation restoration measures investigated in this study significantly increased the content and storage of SOC. The NT significantly increased the levels of EOC, DOC, MBC, and LFOC, while YC notably increases EOC content and SL also markedly boosts LFOC. Each vegetation restoration measures exhibited a pronounced “surface aggregation effect” on soil carbon pools. The CPMI exceeded 100% in the 0–40-cm soil layer but fell below 100% in the 40–60-cm layer. This indicates that vegetation restoration measures significantly enhanced the quality of the carbon pool in surface and shallow soils but not in deeper layers. Among the vegetation restoration measures, NT and SL significantly increased the activities of BG, CBH, SUC, and PPO. Both correlation analysis and redundancy analysis (RDA) confirmed that soil enzyme activity is the dominant factor explaining variations in soil labile organic carbon fractions and the CPMI. The Partial Least Squares Path Model (PLS-PM) revealed a synergistic vegetation–nutrients–enzymes–carbon pool mechanism, whereby vegetation restoration enhances soil nutrients (e.g., TN and TP), stimulates hydrolytic and oxidative enzyme activities, and thereby regulates labile carbon fractions and CPMI. Overall, when restoring vegetation in areas affected by desertification, *Caragana korshinskii* should be prioritized as a restoration species, as it can effectively promote the recovery of soil ecological functions and improve the quality of carbon pools in desert regions. Future research could establish time series with varying restoration durations, conduct seasonal sampling, and incorporate analyses of microbial community structure to provide a more comprehensive understanding of the mechanisms underlying the evolution of soil carbon sink functions during vegetation restoration in desertified areas.

## Data Availability

The raw data supporting the conclusions of this article will be made available by the authors, without undue reservation.
